# Detoxification of the Mycotoxin Citrinin by a Manganese Peroxidase from *Moniliophthora roreri*

**DOI:** 10.3390/toxins14110801

**Published:** 2022-11-18

**Authors:** Shuai Wang, Xiaolu Wang, Huoqing Huang, Tao Tu, Huiying Luo, Yuhong Zhang, Bo Liu, Bin Yao, Wei Zhang, Xiaoyun Su

**Affiliations:** 1 Biotechnology Research Institute, Chinese Academy of Agricultural Sciences, Beijing 100081, China; 2 State Key Laboratory of Animal Nutrition, Institute of Animal Sciences, Chinese Academy of Agricultural Sciences, Beijing 100193, China

**Keywords:** citrinin, manganese peroxidase, mycotoxin, red yeast rice, detoxification

## Abstract

Citrinin (CIT) is a mycotoxin found in foods and feeds and most commonly discovered in red yeast rice, a food additive made from ordinary rice by fermentation with *Monascus*. Currently, no enzyme is known to be able to degrade CIT effectively. In this study, it was discovered that manganese peroxidase (*Mr*MnP) from *Moniliophthora roreri* could degrade CIT. The degradation appeared to be fulfilled by a combination of direct and indirect actions of the *Mr*MnP with the CIT. Pure CIT, at a final concentration of 10 mg/L, was completely degraded by *Mr*MnP within 72 h. One degradation product was identified to be dihydrocitrinone. The toxicity of the CIT-degradation product decreased, as monitored by the increased survival rate of the Caco-2 cells incubated with *Mr*MnP-treated CIT. In addition, *Mr*MnP could degrade CIT (with a starting concentration of up to 4.6 mg/L) completely contaminated in red yeast rice. *Mr*MnP serves as an excellent candidate enzyme for CIT detoxification.

## 1. Introduction

Mycotoxins, produced by certain kinds of fungi, refer to a large number of secondary metabolites with varying structures. Citrinin (CIT), a polyketide mycotoxin, was first isolated from a *Penicillium citrinum* culture in the 1930s and is now known to be mainly produced by the *Monascus*, *Aspergillus*, and *Penicillium* genera [1]. Although antibacterial and neuroprotective effects have been documented for CIT [2], many studies in the past few years have undoubtedly demonstrated that CIT majorly exert negative effects [3]. For example, CIT disrupts the distribution and function of organelles of mouse oocytes and significantly reduces their developmental ability [4]. Collectively, the many in vitro and in vivo studies provide strong evidence supporting the reproductive toxicity, teratogenicity, and embryotoxicity of CIT [5]. In 2019, the European Union amended Section 2.8.1 of the Annex to Regulation (EC) 1881/2006 to provide a maximum legislative level of 100 μg/kg for CIT in food supplements based on rice fermented with red yeast *Monascus purpureus* [6,7]. In China and Japan, the maximal limit of CIT in red yeast rice is 50 and 200 μg/kg, respectively [8]. CIT can also be found in feed: in an analysis of 90 chicken and pig feed samples from Belgium, nearly 50% of the samples have been discovered to be contaminated with CIT, with the highest concentration being 3.9 μg/kg [9]. In Russia, an analysis of 1743 feed samples indicated the highest incidence of 30% of CIT contamination in sunflower meal, with the highest concentration of 998 μg/kg discovered in barley [10]. Note that CIT in animal feed can be harmful to humans through accumulation in the food chain [11].

The widespread contamination of the harmful CIT requires elimination of this mycotoxin in foods and feeds. To remove CIT contamination, physical, chemical, and biological treatments have been used. Treatment at a high temperature, such as at 140 °C, turns CIT into CIT H1. However, the toxicity of this compound was even higher than that of CIT [12]. Some specific magnetic nanoparticles have been used to adsorb CIT in *Monascus*-fermented products [13]. In addition, CIT can be degraded by chemical method. A phosphate-ethanol mixture was reported to remove 91.6% of CIT from *Monascus* rice within 70 min [14]. In addition to degradation, chemical treatment can also inhibit CIT production. For example, adding flavonoids, such as troxerutin, inhibited CIT production by 53.7–87.9% [15]. It is worth noting that all of these methods, being expensive, do not completely remove CIT. These shortcomings have prompted the seeking of other efficient, safe, and environmentally friendly ways, such as microbial and enzymatic treatments to decompose CIT.

To date, the micro-organisms that are reported to be able to degrade CIT effectively include *Cryptococcus podzolicus* Y3 [16], *Rhizobium borbori* [17], and *Klebsiella pneumonia* [18], with the decomposition rate reaching as high as 97.9% [18]. However, note that in microbial treatment, the presence of a large number of residual cells and high concentrations of metabolites after treatment may change the final quality of the product. It is noted that the key elements involved in microbial detoxification are actually the enzymes. Enzymes appear to be superior to microbes in that there are no unwanted cells or their metabolites after treatment. However, even in CIT-degrading bacteria, the enzyme(s) responsible for eliminating CIT have not been clearly identified, thus hindering the use of enzymes to detoxify CIT.

Manganese peroxidase (MnP) is a special heme-containing peroxidase that can oxidize phenolic lignin molecules via direct interaction or non-phenolic lignin molecules through the assistance of Mn^3+^ [19]. The ability of MnP to detoxify a variety of feed and food mycotoxins, including aflatoxin, zearalenone, deoxynivalenol, fumonisin, and patulin, have been demonstrated before [20]. CIT is a small mycotoxin and bears a phenolic hydroxyl group, appearing to be a suitable substrate of MnP. However, the ability of MnP to degrade CIT is still unclear. Therefore, in order to know whether manganese peroxidase can degrade CIT and whether this degradation leads to detoxification, in this study, the ability of a manganese peroxidase *Mr*MnP from *Moniliophthora roreri* [21] to degrade CIT was explored. One degradation product, i.e., dihydrocitrinone, was identified by mass spectrometer and the toxicity of the degradation products was determined. In addition, red yeast rice, a *Monascus*-fermented rice product popular in Asia as a food staple, was used as a model to investigate if the *Mr*MnP-catalyze CIT degradation would be affected by possible interfering compounds in the foods.

## 2. Results and Discussion

### 2.1. Effect of the Buffer Used on CIT Degradation by MrMnP

In order to test the ability of manganese peroxidase to inactivate CIT, *Mr*MnP was heterologously produced in *Pichia pastoris* and the recombinant protein was affinity purified and then incubated with CIT in different buffers. The buffer system is well-known to largely affect the manganese peroxidase-catalyzed degradation of mycotoxins, such as patulin. In this study, six different buffers were tested and they included acetate (monocarboxylic acid), malonate and oxalate (dicarboxylic acid), citrate (tricarboxylic acid), lactate (α-hydroxy carboxylic acid), phosphate (inorganic acid) and MES and HEPES (amphoteric ion buffers, which represent 2-morpholine ethanesulfonic acid and represent 2-[4-(2-hydroxyethyl)-1-piperazinyl] ethanesulfonic acid, respectively).

**Figure 1:**
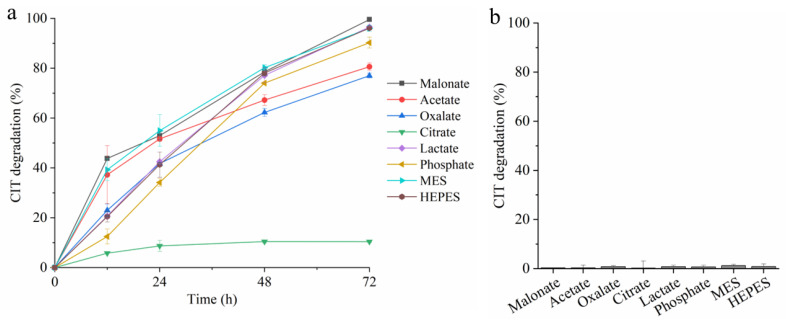
Effects of the buffer components on CIT degradation. (**a**) Degradation of CIT by *Mr*MnP in presence of various buffers. (**b**) Degradation of CIT when no *Mr*MnP was added. The reactions were carried out by incubating 10 mg/L of CIT and 500 U/L with or without *Mr*MnP in one of the buffers containing malonate, acetate, oxalate, citrate, lactate, phosphate, MES, and HEPES at 30 °C for 72 h. The data shown is the average ± standard deviation.

Unlike patulin, the degradation of CIT as unveiled in a time-course analysis in the malonate, lactate, phosphate, MES, and HEPES buffers were very similar, gradually growing to a rate of above 90% (90.2 ± 2.2%~100%, Figure 1a) at the end of the reaction. In acetate and oxlate buffers, the degradation was slightly reduced to 80.6 ± 1.5% and 77.0 ± 0.9, respectively. Although the Mn^3+^ generated during the oxidation of Mn^2+^ by *Mr*MnP can chelate with malonate and oxlate to form stable complexes, it could not chelate with acetate, phosphate, MES, and HEPES. This suggested that CIT degradation could be through a direct interaction of the enzyme with the substrate. It was also possible that, in CIT degradation, the oxidized Mn^3+^ itself, but not the Mn^3+^-acid chelate and the subsequently generated radicals generated from the organic acids, could rapidly react with this mycotoxin. The lowest degradation rate (10.4 ± 0.5%) at 72 h was observed for the citrate buffer. After 72 h of incubation, *Mr*MnP still remained a residual enzyme activity of 190 U/L. The pKa for each of the buffers used in the study is: malonate, 2.8 and 5.7; acetate, 4.7; oxalate, 1.3 and 4.3; citrate: 3.1, 4.8, and 6.4; lactate: 3.9; phosphate: 2.2, 7.2, and 12.3; MES: 6.1; HEPES: 7.6. Therefore, it is hard to notice a strong relationship of the pKa of a buffer with the ability of *Mr*MnP to degrade citrinin. It is thus postulated that other mechanisms (such as effect on *Mr*MnP’s stability, etc.) might affect the ability of *Mr*MnP to degrade citrinin. Note that when no *Mr*MnP was added, there was almost no degradation of CIT observed (Figure 1b).

Previously, we have shown that the composition of the buffer solution affects the efficiency of manganese peroxidase. In the study for the four major feed mycotoxins (aflatoxin B_1_, zearalenone, deoxynivalenol, and fumonisin B), the used buffers were malonate, acetate, and lactate [20]. In a more recent study focusing on degradation of patulin, six different buffers (the same as above) were tested [22]. Note that in these buffer solutions, *Mr*MnP acted most efficiently in the malonate buffer, suggesting that this dicarboxylic acid was involved in the reaction.

### 2.2. Effect of Mn^2+^ on CIT Degradation by MrMnP

**Figure 2:**
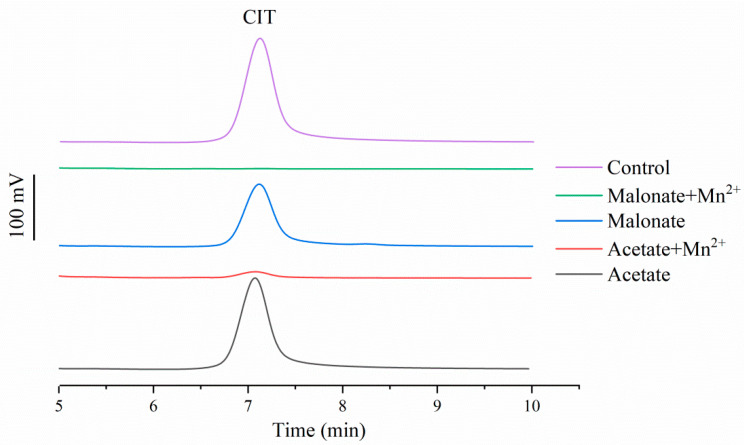
Mn^2+^ played an important but not exclusive role in degrading CIT. The reactions were carried out by incubating 500 U/L of *Mr*MnP with 10 mg/L of CIT in the acetate or malonate buffer either in presence or in absence of Mn^2+^ at 30 °C for 72 h. Then, the reaction products were analyzed by HPLC. The data demonstrated in the figure were prepared by integrating the HPLC traces from analyses of multiple reactions. The bar on the left represents a signal of 100 mV.

To further decipher the mechanisms involved in CIT degradation, the effect of Mn^2+^ on *Mr*MnP-catalyzed degradation of CIT was investigated. Normally, a manganese peroxidase has two substrate channels. One is the δ-heme edge, which is responsible for oxidizing hydrophobically bound substrates, such as ABTS and phenolic compounds. The other is the γ-heme edge, which is involved in the catalysis of Mn^2+^ [23]. Therefore, *Mr*MnP was incubated with CIT either in presence or in absence of Mn^2+^. The degradation rates of CIT were low (but not disappeared) in both malonate (15.6 ± 1.0%) and acetate (5.1 ± 1.5% in acetate) buffers without Mn^2+^. Complete degradation of CIT was observed only in the presence of malonate and Mn^2+^ (Figure 2). This suggested that two mechanisms could be involved in CIT degradation: first, a minus portion of CIT was degraded through direct interaction of the enzyme with the substrate, representing the observed low degradation rate in absence of the Mn^2+^; second, the major part of degradation appeared to be still Mn^2+^-dependent, although it appeared that this mechanism could be through direct action of Mn^3+^, as well as through the Mn^3+^-dicarboxylatechelate and the carboxylate-derived radicals. Therefore, both the δ-heme and γ-heme edges appeared to be involved in CIT degradation.

### 2.3. CIT Was Detoxified after MrMnP Treatment

**Figure 3:**
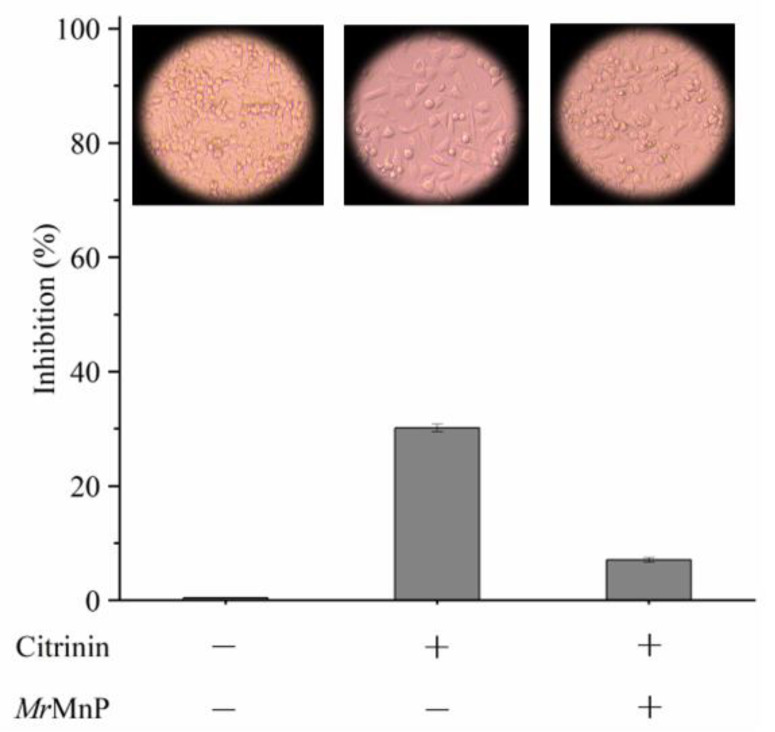
*Mr*MnP treatment of CIT led to detoxification as determined by using Caco-2 cell as a model. The cells were sub-cultured in 96-well plates with an inoculation density of 1 × 10^5^ cells per well. Before mycotoxin treatment, the cells were left for adhesion to the wall for 24 h. CIT was added to cells at a concentration of 10 mg/L. Degradation of CIT was achieved by treating with *Mr*MnP for 72 h. Then, the culture was continued for 48 h. Each value in the panel represents the mean ± SD (*n* = 6).

To use *Mr*MnP for CIT detoxification, the degradation products need to be less toxic. One well-known toxicity of mycotoxins to humans and animals is that they can cause damage to intestinal physiological function by destroying intestinal cells [24]. Caco-2 is a kind of human colon adenocarcinoma cell, which is similar to differentiated intestinal epithelial cells in structure and function. Therefore, it has been successfully used as a model cell line to monitor the toxicity of CIT and its degradation products [25]. The 3-(4-dimethylthiazolyl-2-yl)-2-diphenyltetrazolium (MTT) method was used to determine the residual toxicity [26]. With this method, 10 mg/L of CIT displayed an obvious toxicity by inhibiting the growth of Caco-2 cells to 30.1 ± 0.6% (Figure 3). When CIT was treated with *Mr*MnP, the inhibition of Caco-2 cell growth was reduced to 7.1 ± 0.4%. All samples were ultrafiltered before being used to treat the Caco-2 cells, thus avoiding direct contact of the *Mr*MnP enzyme with the cells. This assay indicated that *Mr*MnP can play a role in detoxifying CIT.

### 2.4. Structural Analysis of the Degradation Products

**Figure 4:**
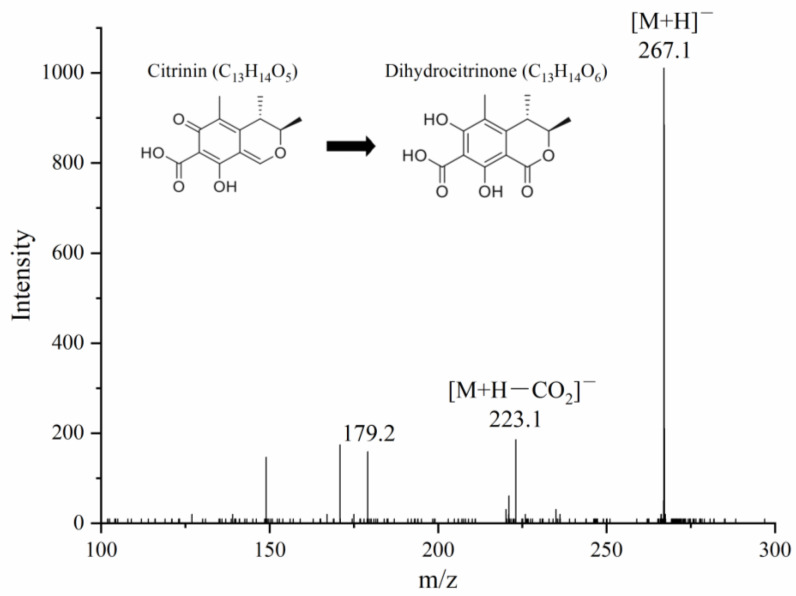
Identification of dihydrocitrinone as one degradation product of CIT. CIT was incubated with 500 U/L of *Mr*MnP in 50 mM malonate buffer (pH 5.0) supplemented with 1 mM MnSO_4_ and 0.1 mM H_2_O_2._ The reaction was carried out at 30 °C for 72 h. The degradation products were analyzed by UHPLC–MS/MS and the secondary mass spectrum of the identified degradation product dihydrocitrinone was shown. In [M+H]^−^, the M was referring to dihydrocitrinone. Its two daughter ions were with *m*/*z* values of 223.1 and 179.2, respectively, reflecting continuous loss of the carbon dioxide from dihydrocitrinone.

The degradation products of CIT as catalyzed by *Mr*MnP were further analyzed by UHPLC-MS/MS. Although absorbance scanning of the reaction product from 190 nm to 400 nm did not reveal an obvious peak, using the UHPLC-MS/MS analysis, dihydrocitrinone (3,4-dihydro-6,8-dihydroxy-3,4,5-trimethyl-isocoumarin-7-carboxylic acid) was identified to be one degradation product. The parent ion appeared at *m*/*z* 267.1 [M+H]^−^, producing daughter ions of 223.1 [M+H−CO_2_]^−^ and 179.2, respectively (Figure 4). The continuous loss of carbon dioxide of the parent ion produced sub-ions [27,28]. In a previous study, Margaret et al. used *Penicillium viridicatum* to degrade CIT and also obtained dihydrocitrinone as one of the degradation products [29]. It is also worth noting that there are studies demonstrating the largely decreased toxicity of dihydrocitrinone in comparison with CIT [30,31,32].

### 2.5. Removal of CIT in Red Yeast Rice

**Figure 5:**
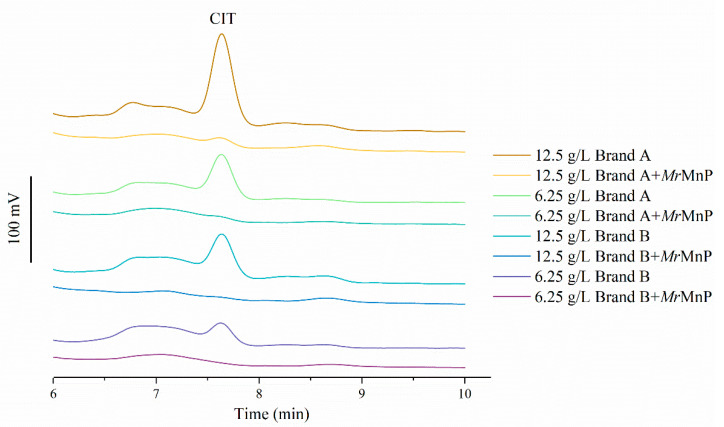
*Mr*MnP could degrade CIT in presence of red yeast rice. Two kinds of commercially available red yeast rice containing different concentrations of CIT (4.6 and 2.1 mg/L of CIT in 12.5 and 6.25 g/L of Brand A, respectively; and 2.4 and 1.2 mg/L of CIT in 12.5 and 6.25 g/L of Brand B, respectively) were used. *Mr*MnP (500 U/L) was incubated with each of the two kinds of red yeast rice and the samples taken out at 72 h were analyzed by HPLC. The data demonstrated in the figure were prepared by integrating the HPLC traces from analyses of multiple reactions. The bar on the left represents a signal of 100 mV.

Some enzymes with the ability to degrade mycotoxins may not effectively degrade mycotoxins in food or feed. This is because food or feed contains many ingredients that can either adsorb mycotoxins or act as competitors or even inhibitors of enzymes. For example, many mycotoxins, including zearalenone and aflatoxin B1, are adsorbed on lignocellulose to a certain extent, and lignin phenols are naturally the substrates of MnPs and may interfere with the degradation of mycotoxins [33,34,35,36,37]. CIT has often been found in some traditional Asian food pigments, such as those made from rice fermented by the fungus *Monascus*. These pigments are commonly used in meat preservation and food coloring [38]. In order to study whether *Mr*MnP can degrade CIT in the real environment with possible interfering ingredients, first CIT was added to different concentrations of *Monascus*-fermented pigment. The *Monascus*-generated pigment did not affect the degradation of CIT (Figure S1). Then, the commercially available red yeast rice containing varying concentrations of CIT was used as the substrate. CIT in red yeast rice (4.6 and 2.1 mg/L of citrinin in 12.5 and 6.25 g/L of Brand A, respectively; and 2.4 and 1.2 mg/L of CIT in 12.5 and 6.25 g/L of Brand B, respectively) could all be degraded by *Mr*MnP within 72 h of incubation (Figure 5), as could be observed from the disappearance of the CIT peak (with a retention time of 7.625 min) in HPLC analysis. Therefore, under the tested conditions, *Mr*MnP can be used as a means to remove CIT in the red yeast rice.

## 3. Conclusions

In this study, it was demonstrated that *Mr*MnP could degrade CIT and the degradation appeared to be through a combination of direct interaction and indirect action involving both the δ-heme and γ-heme edges. *Mr*MnP degraded CIT to dihydrocitrinone and possibly other unidentified chemicals and the mycotoxin’s toxicity largely decreased after the enzyme treatment. Intriguingly, *Mr*MnP-catalyzed degradation of CIT (up to 4.6 mg/L) was not affected by the red yeast rice. While the presented data pinpoint to the potential of using this enzyme to detoxify CIT contamination in such pigment-containing foods, one should also be cautious of the manganese introduced in the reaction, which, however, could be minimized by deliberately designed sample processing (such as extensive washing).

## 4. Materials and Methods

### 4.1. Strains and Plasmids

The strain used for gene cloning was *Escherichia coli* Trans1-T1 (TransGen, Beijing, China). *Moniliophthora roreri* is a manganese peroxidase-producing filamentous fungus. One of the MnPs (i.e., *Mr*MnP used in this study) can be successfully recombinantly expressed in *Pichia pastoris* [21,22]. The plasmid used to construct the expression vector was pPICZα(A) (Invitrogen, Carlsbad, CA, USA). *Pichia pastoris* X-33 (Invitrogen, Carlsbad, CA, USA) was used for recombinant expression of the *Mr*MnP, and it was routinely cultured and maintained on YPD-agar plates (where YPD represents yeast potato dextrose, containing 1% yeast extract, 2% peptone, 2% glucose). A single colony of *P. pastoris* strains was inoculated in the YPD liquid medium in a shaker flask and cultured at 30 °C.

### 4.2. Expression and Purification of MrMnP

The construction of the plasmid for expressing *MrMnP* was reported earlier [22]. Briefly, the *MrMnP* manganese peroxidase gene (GenBank accession number: ESK95360.1) was synthesized by the GenScript Biotech Corp. (Nanjing, China) and ligated to the pPICZα(A) vector by *Eco*RI and *Not*I sites and then the *Dra*I-linearized recombinant expression vector was introduced into *P. pastoris* X33 competent cells by electroporation. Positive transformants were screened on YPD agar plate containing 100 μg/mL of zeocin. The transformants carrying *MrMnP* gene were cultured in BMGY liquid medium containing 1% glycerol until OD_600_ reached 6. The culture was centrifuged (1200× *g*, 25 °C, 5 min) and then suspended in BMMY medium containing 1% methanol. Methanol at a final concentration of 1% (*v*/*w*) was added every 24 h to induce the production of recombinant manganese peroxidase.

The *Mr*MnP recombinant protein was purified from the fermentation broth by using a Ni-NTA column, according to manufacturer’s instructions (QIAGEN, Dusseldorf, Germany). Briefly, the column was equilibrated with 30 mL of binding buffer (20 mM phosphate buffer, 0.5 M NaCl, pH 7.4). A total of 20 mL of crude enzyme solution was then passed through the purification column. Impurity proteins were removed with 50 mL of the washing buffer (20 mM phosphate buffer, 0.5 M NaCl, 20 mM imidazole, pH 7.4). Finally, the proteins were eluted with elution buffer (20 mM phosphate buffer, 0.5 M NaCl, 0.2 M imidazole, pH 7.4).

### 4.3. Degradation of CIT by MrMnP

In order to determine the ability of *Mr*MnP to degrade CIT, the manganese peroxidase activity was first calibrated with 2,2′-azino-bis (3-ethylbenzothiazoline-6-sulfonic acid) (ABTS), which was carried out by monitoring the oxidation of ABTS (ε_420_ = 36,000 M^−1^cm^−1^) in a buffer containing 50 mM malonate, 1 mM ABTS, 1 mM MnSO_4_ and 0.1 mM H_2_O_2_ (pH 5.0 and 30 °C) at 420 nm as described in Qin et al. [39]. Then, *Mr*MnP with an activity of 500 U/L (equaling to 3.3 mg/L of the recombinant protein) against ABTS was incubated with 10 mg/L of CIT in 50 mM malonate buffer, 1 mM MnSO_4_, and 0.1 mM H_2_O_2_. The reaction was carried out at 30 °C. At the end of the reaction, 3 volumes of methanol were added to the mixture to terminate the reaction, and the reaction products were analyzed by high performance liquid chromatography (HPLC).

### 4.4. Effect of the Buffer Systems on Degradation of CIT by MrMnP

To further determine the effect of buffer composition on the degradation of CIT, malonate (50 mM) was replaced by different buffers at the same concentration, which included acetate, lactate, citrate, oxalate, phosphate, MES, and HEPES. In all the reaction systems tested, the pH value of the buffer was adjusted to 5.0. The reaction was carried out at 30 °C for 72 h and then the products were analyzed by HPLC.

### 4.5. Effect of Mn^2+^ on Degradation of CIT

In order to decipher the mechanisms involved in degradation of CIT, the effect of Mn^2+^ on the degradation rate of CIT in malonate and acetate systems was determined. All the reactions were carried out at 30 °C for 72 h and then the samples were taken out for to analysis in HPLC.

### 4.6. Toxicity Assay

The human colon cancer Caco-2 cell line (ATCC HTB-37) was purchased from ATCC (Rockville, Maryland). The culture medium included Dulbecco’s modified Eagle’s medium (DMEM, Gibro) supplemented with 10% heat inactivated fetal bovine serum (Gibro), 2 mM L-glutamine (Sigma-Aldrich, St. Louis, MO, USA), 100 mg/L penicillin, and 100 g/mL streptomycin (Gibro). The cells were incubated with 100% relative humidity and 5% CO_2_ at 37 °C. The cells were sub-cultured in 96-well plates with an inoculation density of 1 × 10^5^ cells per well. Before mycotoxin treatment, the cells were left for adhesion to the wall for 24 h. CIT was diluted to 10 mg/L in the culture medium and added to cells. Then, the culture was continued for 48 h. The cells added with medium only were used as control.

The viability of Caco-2 cells treated with mycotoxins and *Mr*MnP-degraded products was evaluated by 3-(4-dimethylthiazolyl-2-yl)-2-diphenyltetrazolium (MTT) colorimetric assay [40]. The rationale is that succinate dehydrogenase in the mitochondria of living cells can reduce exogenous MTT to water-insoluble blue-purple formazan and deposit in the cells, while dead cells do not have this function. DMSO can dissolve formazan in cells with a maximal optical absorption at the wavelength of 490 nm. At the end of incubation, 10 μL of MTT with a final concentration of 5 mg/mL was added to each well and placed in a moisturizing incubator at 37 °C for 4 h. The survival rate of the control group was set as 100%. The cell death rate (%) was calculated as (OD_ck_ − OD_sample_)/OD_ck_ × 100%, where OD_ck_ and OD_sample_ were the optical densities of the control and sample cells as measured at 490 nm, respectively.

### 4.7. HPLC and LC-MS/MS Analyses

The HPLC analysis of CIT (with a detection limit of 5 μg/L) was carried out using a SHIMADZU 20A series instrument (Kyoto, Japan). The chromatographic column was Agilent ZORBAXSB-C18 (5 μm, 4.6 mm × 250 mm). The column temperature was set to 30 °C. The mobile phase A with a pH of 3.2 is water with 0.1% acetic acid and the mobile phase B is acetonitrile. The elution used 65% mobile phase B with a flow rate 0.75 mL/min. The fluorescence detector was used with the excitation wavelength at 331 nm and the emission wavelength at 500 nm.

To elucidate the nature of the degradation products of CIT, LC-MS/MS (with a detection limit of 10 ng/L for CIT) was further utilized, which was carried out by coupling a SHIMADZU Nexera UHPLC system (Kyoto, Japan) to an AB-SCIEX 5600+ Triple TOF mass spectrometer (Waltham, MA, USA). The chromatographic column was XBrige BHE C18 (2.5 μm, 2.1 mm × 150 mm), the column temperature was 40 °C, the mobile phase A was acetonitrile, and the mobile phase B was 0.1% formic acid. The injection volume was 1 µL and the flow rate was 0.3 mL/min. The elution procedure was as follows: initial 50% phase A; 7.0 min, 100% phase A; 9.0 min, 100% phase A; 10.0 min, 50% phase A; 12.0 min, 50% phase A. The detection conditions of mass spectrometry were as follows: positive ion; TOF-mass (Da) 100–800; ion source: Duo Spray Ion Source; ion source gas 1:50; ion source gas (nitrogen) 2:50; curtain gas (nitrogen): 25; temperature: 450, IonSpray voltage floating (ISVF): 5500; declustering potential: 60.0; collision energy: 35.0; accumulation time: 0.1 s. ion scanning conditions: declustering potential: 60.0; collision energy: 35.0; collision energy spread: 15.0; ion release delay: 67; ion release width: 25.

### 4.8. Determining CIT Degradation by MrMnP in Red Yeast Rice

The *Monascus*-fermented pigment and red yeast rice were obtained from a local retail market. CIT reference material (Sigma-Aldrich, St. Louis, MO, USA) with a purity >98% was used to spike *Monascus*-fermented pigment at a final concentration of 5 mg/L. CIT was, however, detected in red yeast rice. The *Monascus*-fermented pigment is produced by submerged fermentation of *Monascus* using the rice and soybean as the carbon sources, followed by extensive extraction. Red yeast rice is a fermentation mixture of steamed rice with *Monascus* and its metabolites. In the presence of 50 mM malonate solution and 1 mM MnSO_4_, *Mr*MnP with a final concentration of 500 U/L was added to the simulated CIT-contaminated pigment and red yeast rice (6.25 g/L and 12.5 g/L, respectively). The reaction was initiated by adding hydrogen peroxide to 0.1 mM. Samples were taken at 0 h and 72 h for HPLC analysis.

### 4.9. Statistical Analysis

At least three repetitions were used in this study. The results were expressed as mean ± standard deviation (SD). The data were analyzed by one-way analysis of variance (ANOVA) using the software SPSS developed by IBM (Version 17.0; IBM, Armonk, NY, USA).

## Data Availability

The data presented in this study are available on request from the corresponding author.

## Supplementary Materials

The following supporting information can be downloaded at: https://www.mdpi.com/article/10.3390/toxins14110801/s1, Figure S1: CIT within the *Monascus-*fermented pigment could be degraded by *Mr*MnP.

## Abbreviations

CIT	Citrinin
MnP	Manganese peroxidases
ABTS	2,2′-Azino-bis (3-ethylbenzothiazoline-6-sulfonic acid)
MTT	3-(4-dimethylthiazolyl-2-yl)-2-diphenyltetrazolium
H_2_O_2_	Hydrogen peroxide
MES	2-Morpholinoethanesulphonic acid
HEPES	2-[4-(2-Hydroxyethyl)-1-piperazinyl] ethanesulfonic acid
