# Supplementary material for: Detoxification of the Mycotoxin Citrinin by a Manganese Peroxidase from Moniliophthora roreri
Source: Toxins (Basel). 2022 Nov 18;14(11):801. doi: 10.3390/toxins14110801 (PMC9693499; doi:10.3390/toxins14110801)
Supplement: Supplementary file 1 [file toxins-14-00801-s001.zip › toxins-1982047-supplementary.pdf]

toxins

MDPI

Supplemental Materials: Detoxification of the Mycotoxin Citrinin by a Manganese Peroxidase from *Moniliophthora roreri*

Shuai Wang, Xiaolu Wang, Huoqing Huang, Tao Tu, Huiying Luo, Yuhong Zhang, Bo Liu, Bin Yao, Wei Zhang and Xiaoyun Su

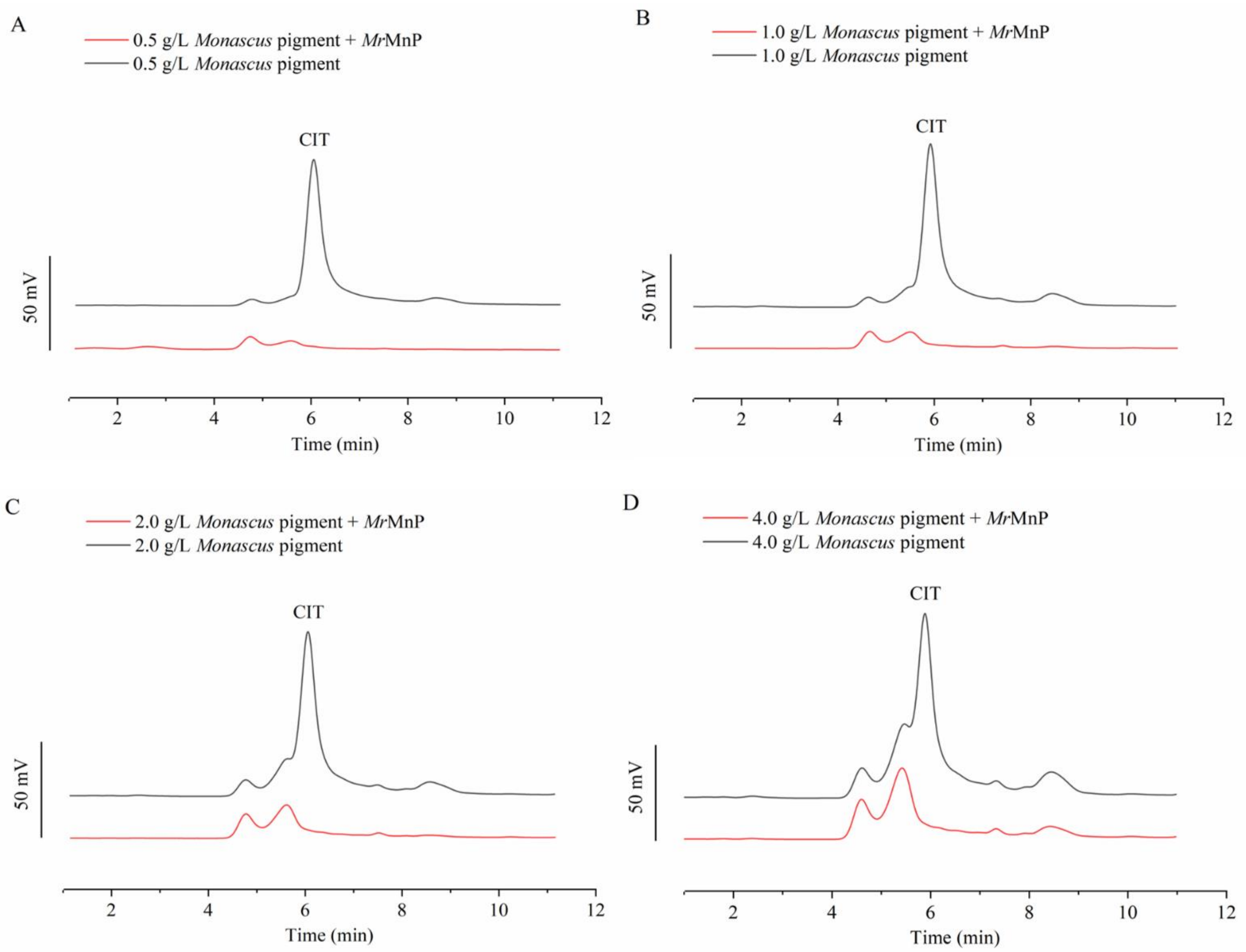

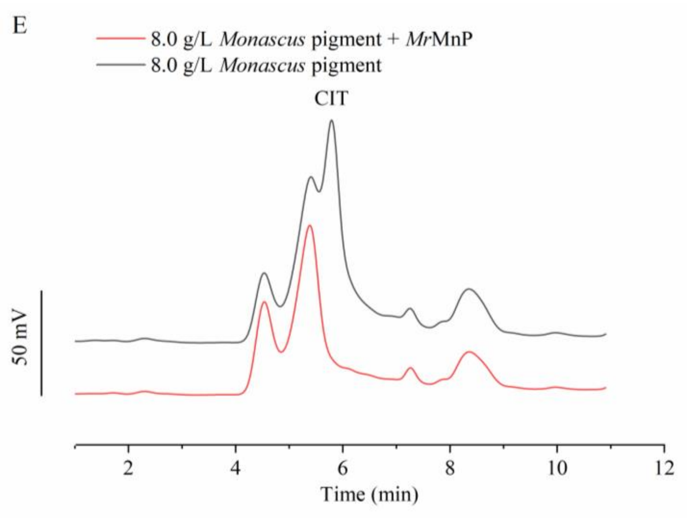

Figure S1. Citrinin within the *Monascus*-fermented pigment could be degraded by *Mr*MnP. Five mg/L of citrinin were added to different concentrations of *Monascus*-fermented pigment. Then 500 U/L *Mr*MnP were added to each of these *Monascus*-fermented pigments and the incubation was carried out for 72 h. The samples were analyzed by HPLC.
